# Carbon Monoxide Induced Erythroid Differentiation of K562 Cells Mimics the Central Macrophage Milieu in Erythroblastic Islands

**DOI:** 10.1371/journal.pone.0033940

**Published:** 2012-03-23

**Authors:** Shlomi Toobiak, Mati Shaklai, Nurith Shaklai

**Affiliations:** 1 Department of Human Genetics and Biochemistry, Sackler Faculty of Medicine, Tel Aviv University, Tel Aviv, Israel; 2 Department of Hematology, Sackler Faculty of Medicine, Tel Aviv University, Tel Aviv, Israel; University of Louisville, United States of America

## Abstract

Growing evidence supports the role of erythroblastic islands (EI) as microenvironmental niches within bone marrow (BM), where cell-cell attachments are suggested as crucial for erythroid maturation. The inducible form of the enzyme heme oxygenase, HO-1, which conducts heme degradation, is absent in erythroblasts where hemoglobin (Hb) is synthesized. Yet, the central macrophage, which retains high HO-1 activity, might be suitable to take over degradation of extra, harmful, Hb heme. Of these enzymatic products, only the hydrophobic gas molecule - CO can transfer from the macrophage to surrounding erythroblasts directly via their tightly attached membranes in the terminal differentiation stage.

Based on the above, the study hypothesized CO to have a role in erythroid maturation. Thus, the effect of CO gas as a potential erythroid differentiation inducer on the common model for erythroid progenitors, K562 cells, was explored. Cells were kept under oxygen lacking environment to mimic BM conditions. Nitrogen anaerobic atmosphere (N_2_A) served as control for CO atmosphere (COA). Under both atmospheres cells proliferation ceased: in N_2_A due to cell death, while in COA as a result of erythroid differentiation. Maturation was evaluated by increased glycophorin A expression and Hb concentration. Addition of 1%CO only to N_2_A, was adequate for maintaining cell viability. Yet, the average Hb concentration was low as compared to COA. This was validated to be the outcome of diversified maturation stages of the progenitor's population.

In fact, the above scenario mimics the *in vivo* EI conditions, where at any given moment only a minute portion of the progenitors proceeds into terminal differentiation. Hence, this model might provide a basis for further molecular investigations of the EI structure/function relationship.

## Introduction

Reticulocytes are formed in specific bone marrow (BM) niches, known as erythroblastic islands (EI). EI are composed of a central macrophage surrounded by erythroblasts in different stages of maturation [1]–[3] and the early presence of these unique structures in fetal liver supports their essential role in erythropoiesis [4], [5]. Bessis and colleagues were the first to characterize the details of EI morphology [2] and suggested that the central macrophages serve as nursing cells for the differentiating erythroblasts. At the terminal differentiation stage, the expelled erythroblast nucleus sweeps hemoglobin (Hb) residues into the macrophage upon engulfment [6] and hence iron was thought to be the nursing material being transferred from the macrophage to erythroblasts [7]. However, intensive research has demonstrated that the source of iron required for Hb Heme synthesis in the BM is from senescent erythrocytes Hb, disintegrated and recycled in the liver and spleen macrophages to be delivered back to the BM by transferrin [8]. In this context, macrophages activity is mediated by heme oxygenase-1 (HO-1) enzyme, responsible for heme catabolism into iron, biliverdin and carbon monoxide (CO) [9]. Thus, identification of the major source of iron for erythropoiesis as originating from liver and spleen macrophages in fact abrogates the putative function of the EI central macrophage as an important iron supplier.

Although HO enzymes exist in all cell types, *a priori*, heme destructive activity in cells, which extensively synthesize Hb, i.e. erythroblasts, appears ambiguous. Indeed, Immenschuh et al. demonstrated that both human BM erythroid precursors and K562 model cells lack expression of the inducible HO-1 and during differentiation, their constitutive HO-2 is depressed [10]. Similarly, another recent study showed that HO-1 has a crucial role in proper erythroblast differentiation even under stress conditions, since reduced HO-1 activity results in disruption of progenitor maturation [11].

An additional characteristic aspect of the EI is the intimate intercellular contact between the central macrophage and differentiating erythroblasts. Strong adhesion occurs during the course of terminal differentiation [3], [10] and subsequently recedes, to facilitate reticulocytes egress from BM niches [12], [13]. Despite extensive research, two central aspects of EI biology are still unclear: (1) Why is adhesion of the erythroblasts to the macrophage strengthened during terminal differentiation? (2) What is the identity of “nursing” material delivered to the surrounding erythroblasts by central macrophage?

Even though there is firm contact between central macrophage and the differentiating erythroblasts, only the hydrophobic gas molecule - CO can freely diffuse via the cellular membranes. Once considered as a HO waste product, CO has been recognized in recent years as a metabolite involved in controlling a variety of physiological and therapeutic activities [14]. This raises the question of how CO and free O_2_ manage not to interfere with each other. Although air is composed of approximately 20% oxygen, in fact free oxygen concentration in the body is low and some BM areas are virtually oxygen devoid [15]. Moreover, it was shown that these conditions play a regulatory role in hematopoietic stem cell maintenance [16]. Hence, lack of oxygen allows other gases to play essential roles in the BM including the EI. This study explored whether CO gas can affect maturation of erythroid progenitors under physiologically relevant conditions prevailing in the BM using K562 cells as a model [17].

## Materials and Methods

### Materials

Nonidet P-40, Trisodium citrate, Trypsin, Trypsin inhibitor, RNase, Hoechst 33342, Propidium-Iodide, Trypan blue, NaCl, Trizma-base, Magnesium acetate, Ficoll- Hypaque, May-Grünwald, Giemsa stain, Ferric citrate, Holo-transferrin (20 µg/ml), 5-Aminolevulinic acid (1 mM) were all purchased from Sigma-Aldrich (Israel).

### Cell culture

#### Media

Cells were grown in RPMI-1640 medium, containing 10% heat-inactivated fetal bovine serum (HI-FBS), 100 U/ml penicillin, 100 µg/ml streptomycin, 1.25 µg/ml nystatin and 2 mM L-glutamine, all obtained from Biological Industries, Beit-HaEmek, Israel. Enriched media: I. For heme synthesis, the following additional nutrients were added to medium: ferric citrate (1 mM), holo-transferrin (20 µg/ml) and 5-aminolevulinic acid (1 mM). II. For globin synthesis, ×2 dose of amino acids solution (Biological Industries, Beit-HaEmek, Israel) was used. “Highly enriched medium”- medium supplemented with heme and globin required nutrients.

#### Cell culture

The K562 cell line, derived from human erythroleukemia cells, was obtained from the American Type Culture Collection (Manassas, VA, USA). Cultures were maintained at 37°C in air containing 5% CO_2_ atmosphere and sub-cultured twice a week at initial concentration of 10^5^ cells/ml. K562 cells (2×10^5^/ml) in exponential growing phase were cultured under different aerobic and anaerobic atmospheres: air (free air exchange containing 5% CO_2_), COA (100% CO), N_2_A (100% N_2_) or 1% COA (i.e. 99% N_2_ and 1% CO). The COA resulted in a concentration of 1 mM in the liquid phase. Throughout the study, the concentration of CO was either 0.01 mM or 1 mM. Both CO and N_2_ -cells were kept is sealed flasks. Proliferation was determined by hemocytometer and cell viability was assessed by Trypan blue (TB) exclusion or propidium-iodide (PI) assay.

### PI penetration assay

Cells were incubated under aerobic and anaerobic atmospheres for 4 days and subsequently washed twice with cold PBS, incubated with 5 µg PI and analyzed by Fluorescence-activated cell sorting (FACS). Nonviable cells were defined as PI positive [18].

### Hb quantification assay

#### Cells lysis

Equal number of cells were taken from each treatment condition, washed twice with cold PBS and lysed (lysis buffer: 140 mM NaCl, 10 mM Trizma-base, 1.4 mM magnesium acetate and 0.5% Nonidet P-40) for 15 min on ice [19]. The lysates were centrifuged for 15 min at 10,000 RPM (5590× *g*). Hb quantification method: We used a recently developed method referred as “difference spectrum” [20] . Briefly, since CO has high affinity to Hb to yield carboxyHb (COHb) and this transfer can be followed by increased and shifted Soret band, the difference spectrum can be used to calculate Hb content in cells without other components and/or light scatter interference. After cell lysis, oxyHb spectrum in cell lysates was monitored by spectrophotometer (GBC, UV/VIS 920) at wavelength range of 350–650 nm. Afterward, CO gas is added and spectrum was measured again. Hb content was calculated from the absorbance of the difference spectrum using the difference extinction coefficient of 78.6 mM/L/cm at 419 nm. Hb content was then divided by number of cells. As it highly probable that cells are not homogeneous in Hb content, this value is referred to average Hb content.

### Glycophorin A (GPA) expression

Cells (5×10^5^) were washed twice with cold PBS and re-suspended in 96 µl PBS with 5% HI-FBS. The cells were then incubated with 4 µl of anti-human PE-GPA antibody (1 µg/ml, BioLegend) in the dark for 20 min at room temperature. After incubation, cells were washed with PBS and the fluorescence was measured by flow cytometry. GPA expression was also observed using fluorescence microscopy.

### Separation of dead cells by Ficoll gradient

Cells were washed and re-suspended in PBS (6 ml). Ficoll-Hypaque (2 ml) was added and the suspension was centrifuged at 125× *g* for 25 min. Viable cells in the supernatant were removed, washed and further processed [21].

### Cell cycle analysis

To determine the DNA content of cells, we used method described by Vindelov et al. [22]. Briefly, after exposure to the different atmospheres, cells (10^6^) were washed once with cold PBS, and re-suspended in 40 mM Citrate/DMSO buffer. After addition of trypsin solution (0.03 mg/ml) for 10 min, trypsin inhibitor (0.5 mg/ml) and RNAse (0.1 mg/ml) were added for another 10 min. Then cells were then stained with PI (0.42 mg/ml) solution for 15 min in the dark, and cell cycle analysis was performed by flow cytometry.

### Morphological analysis

#### Giemsa staining

3×10^5^ cells from each atmospheric condition were washed twice with cold PBS. The cells were then cyto-centrifuged (Cytospin 2, Shandon, Pittsburgh, USA) onto a slide at 200× *g* for 7 minutes and stained with May-Grünwald-Giemsa solution. Cells were analyzed using light microscopy.

#### Scanning electron microscopy (SEM)

Samples were fixed with 2.5% glutaraldehyde in PBS, washed, dehydrated in graded ethanol solutions and dried [23], [24]. Samples were then coated with gold (Polaron SEM coating unit E5100) and examined with a Jeol JSM 840A SEM.

#### Enucleation assay

Cells were double stained with antibodies against GPA (see GPA expression section) and Hoechst 33342 (10 µg/ml) for 20 min in the dark (DNA staining). Cell were washed once with PBS and monitored by fluorescence microscopy.

## Results

**Figure 1:**
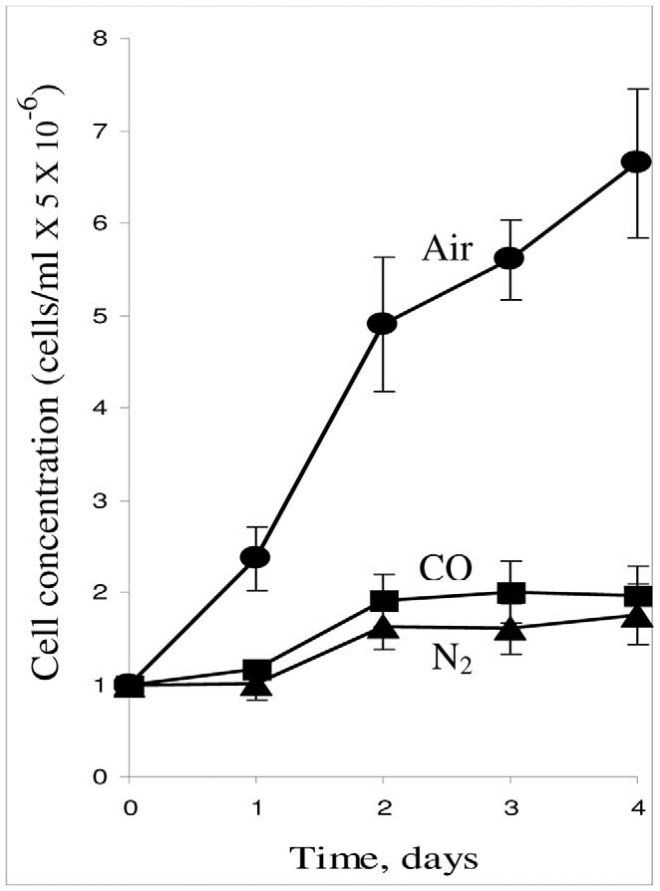
Anaerobic atmospheres inhibit cell proliferation. Cells were incubated under aerobic and two types of anaerobic atmosphere conditions, N_2_A or COA. Cells were counted daily by hemocytometer. Air •–––•; COA ▪–––▪; N_2_A ▴----▴ . Data represent mean ± SD of 4 independent experiments.

To establish if CO has any specific effect on K562 erythroid progenitor cells, we first compared the cellular growth rate under air (referred to as air-cells) and under two anoxic atmospheres, namely COA and its inert gas control N_2_A (referred to as CO-cells and N_2_-cells, respectively). Figure 1 depicts the cellular growth curves under these three atmospheres. As seen, air-cells demonstrate exponential growth. In contrast, under anoxic conditions, only a mild increase in cell proliferation was observed during the first two days, but from this point on, a proliferation plateau was observed; both the CO and N_2_ curves appear virtually the same, within their respective SDs.

**Figure 2:**
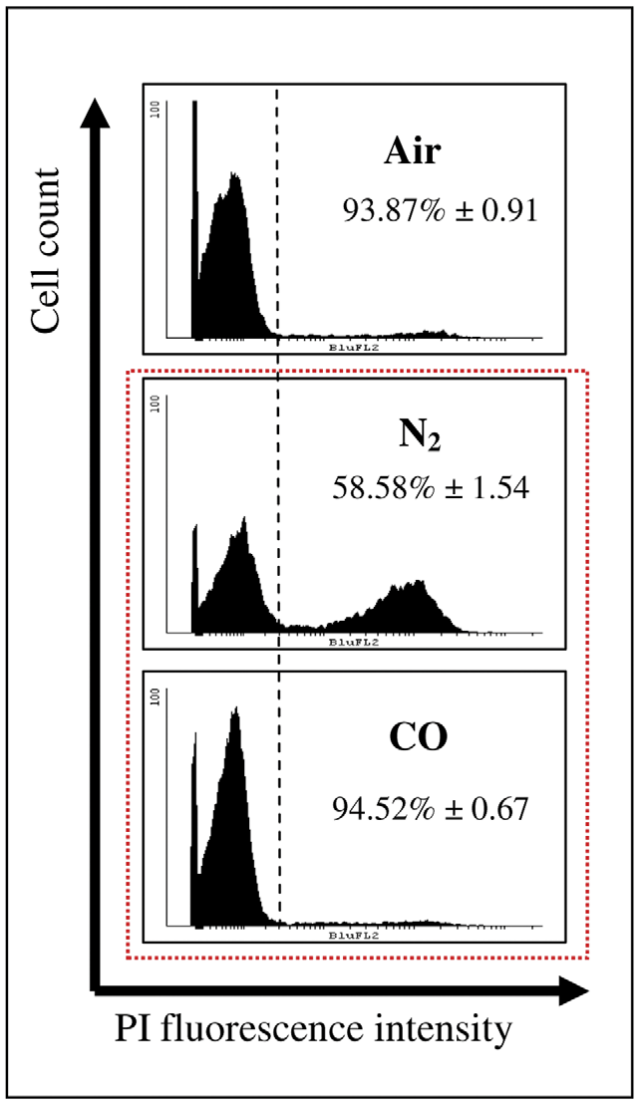
COA but not N_2_A preserves cell viability. Cells were incubated under aerobic and two types of anaerobic atmosphere conditions, N_2_A or COA for 4 days, after which viability was measured via FACS analysis using PI penetration assay. Viability measurements (mean ± SD) from 3 independent experiments are shown.

In order to analyze the reasons for growth inhibition under free oxygen lacking conditions, the cell viability was further determined via PI staining and FACS analysis. It was found that while N_2_-cells lost their viability after extended incubation (4 days), CO-cells preserved their viability, as shown in Figure 2. TB assessment of cell viability also indicated a reduction in viable cells under N_2_A (69.44%±3.24) compared to COA (87.66%±3.82).

**Figure 3:**
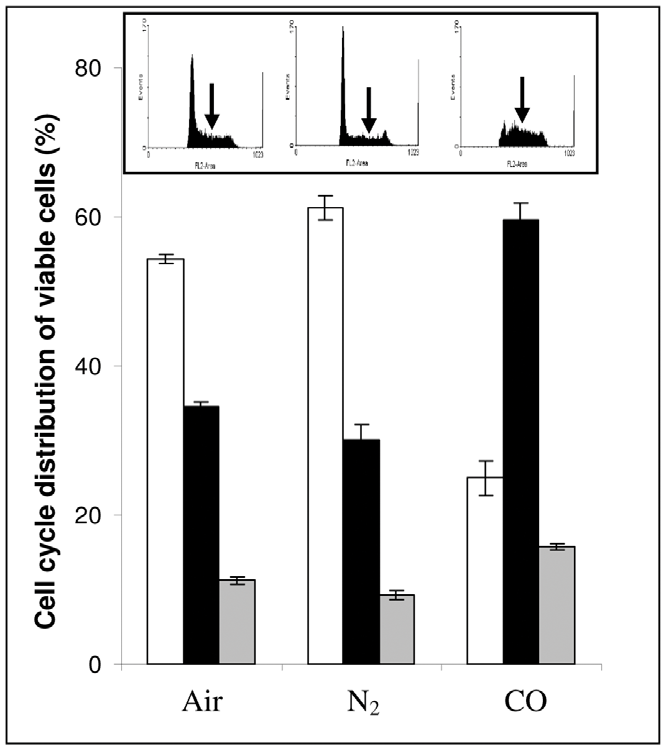
COA, but not N_2_A, results in S-phase arrest of cell cycle. Cells were incubated under aerobic and anaerobic conditions, N_2_A or COA, for 4 days. Insert: representative FACS analysis of cell cycle for K562 cells incubated under different atmospheres. Arrows indicate center of S-phase. Main Figure: phase distribution of viable cells. Mean ± SD from 3 independent experiments is shown. Empty bars - G_0_/G_1_ phase, black bars - S phase and grey bars - G_2_/M phase.

To further clarify the observation of COA induced inhibited proliferation without death, cell cycle analysis was performed on cells incubated for 4 days under air as well as the two anoxic atmospheres. Figure 3 insert illustrates representative examples of cell cycle analysis in each case. As can be seen, air- and N_2_-cells distributed similarly between the cell cycle phases, while in CO-cells, the S-phase fraction is significantly increased. This S-phase arrest was accompanied by a decrease in G0/G1 phases. The average phase distribution of cells incubated under aerobic and anaerobic conditions is shown in main part of Figure 3.

**Figure 4:**
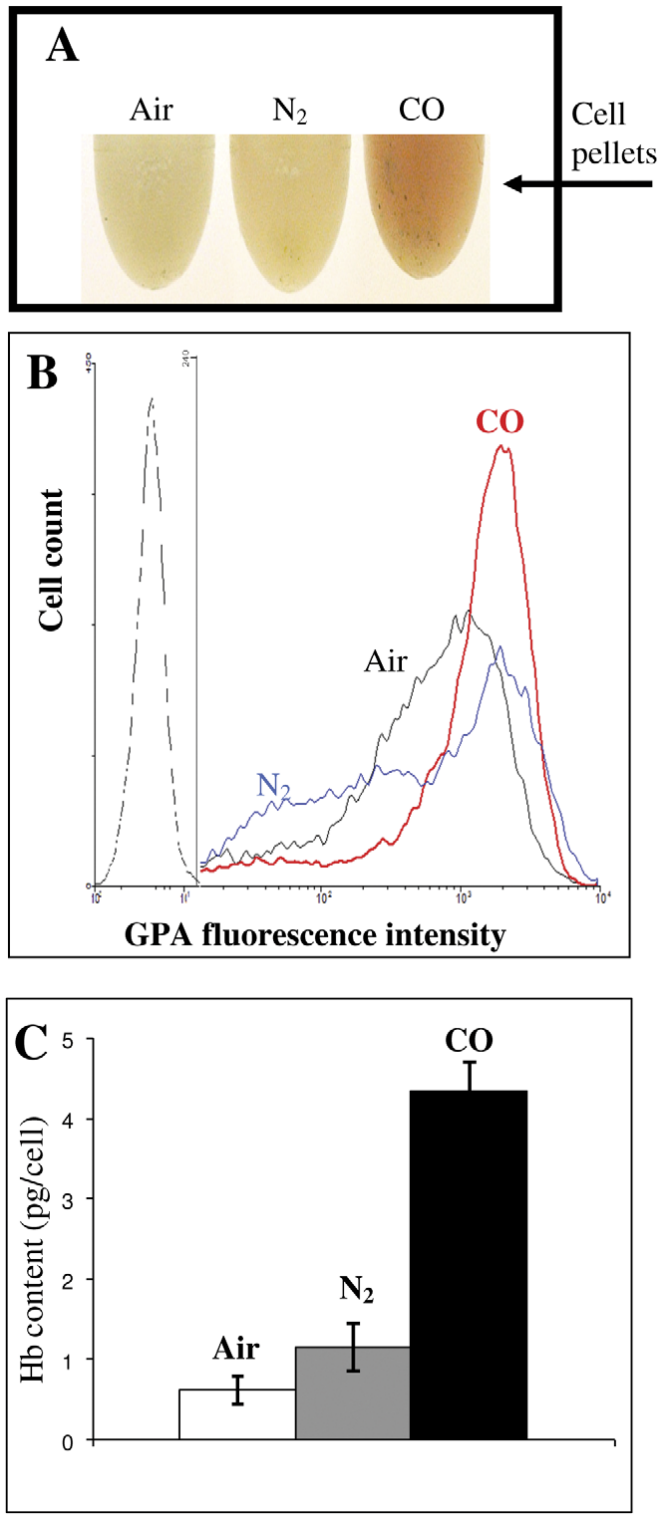
COA induces erythroid differentiation demonstrated by GPA expression and Hb synthesis. Cells were incubated under aerobic and anaerobic conditions for 4 days. (**A**) Cell suspensions were centrifuged and pellets were photographed. Note the prominent red color in the CO-cell pellet. (**B**) FACS analysis of GPA expression. Dashed line: auto-florescence, black - air, blue - N_2_A and red - COA. Note the scale differences on the Y-axis. (**C**) Average cellular Hb content under different conditions using “Hb quantification assay” (see Material and Methods for details). Mean ± SD from 3 independent experiments are shown.

Previous literature pointed to a link between K562 erythroid differentiation and cell cycle S-arrest [25], [26]. Therefore, we hypothesized that the S-arrest imposed by COA could be a result of inhibited proliferation accompanied by maintenance of full viability (Figs. 1 & 2) which may correlate with cellular differentiation. Indeed, as can be seen from the cell-pellet color (Fig. 4A), CO-cells appear to produce a significant amount of Hb as compared to either air or N_2_-cells. GPA, a late erythroid differentiation marker, was used for quantitative analysis of differentiation [27]. Figure 4B illustrates a significant increase in GPA expression in the CO-cells, thus indicating an advanced stage of differentiation. These results also imply that the N_2_-cell population is composed of several sub-populations with higher and lower GPA expression as compared to air. Fractionation of these cells by Ficoll-Hypaque gradient assay (see Materials and Methods) revealed that cells with low GPA expression are mostly dead (data not shown).

Based on these initial results, a refined analysis of erythroid differentiation was carried out using a recently developed method [20]. Figure 4C displays the average Hb content of cells incubated under aerobic and anaerobic conditions. As can be seen, CO-cells has a significantly higher Hb content as compared to air- and N_2_-cells.

**Figure 5:**
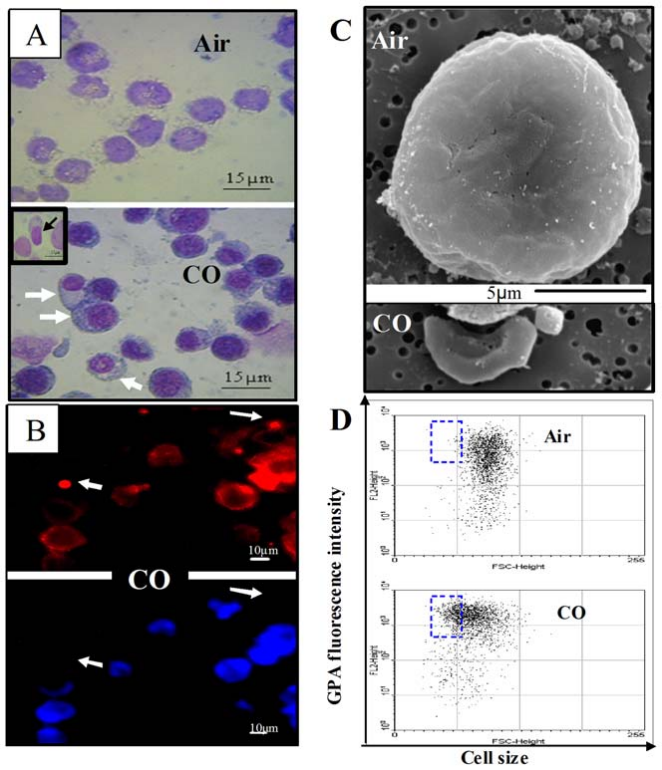
COA induces morphological changes typical of different stages of erythroid maturation. Cells were incubated for 4 days under air or COA. (**A**) Cytospin slides of cells stained with Giemsa-May-Grünwald. Arrows point to COA-cells in various maturation stages. Note the nuclei positioned toward the cell periphery and their condensation. Insert: terminal differentiation as seen by enucleation. (**B**) Cells were double stained with Hoechst 33342 (blue) and α-GPA (red) and then examined by fluorescence microscopy. Arrows indicate the positive GPA and negative Hoechst stained enucleated cells. (**C**) Cell morphology by Scanning Electron Microscopy (SEM). Air panel - a typical immature cell is shown, COA panel - cell size and concave shape typical for erythrocyte. (**D**) FACS analysis of GPA expression and cell size (sample of 10,000 cells). Square-enclosed parameters typical of differentiated of erythroid cells: small diameter due to condensation and population expressing high level of GPA.

Typical, well-characterized morphological changes occurring throughout erythroid differentiation include cell shrinkage, nuclear condensation and peripheral shift, increased cytoplasm/nucleus ratio and eventually enucleation [6], [28]. Hence, as a next step, we examined the morphological changes induced by CO via several techniques. Figure 5A shows standard May-Grünwald-Giemsa staining of cells kept under air or COA. The homogeneous morphology of air-cells coincides with normal immature K562 cells, where most of cell volume is occupied by the nucleus. In contrast, COA population appeared to be composed of both immature as well as more mature cells in various differentiation stages, as shown by their degree of nuclear condensation, peripheral position of nuclei, and increased cytoplasm/nucleus ratio (see Fig. 5A arrows). Progression of cells towards maturation is well evidenced by enucleation (Fig. 5A insert). In order to more accurately assess the enucleation process, additional techniques were employed. Figure 5B illustrates CO-cells double stained with anti-GPA antibodies (upper panel - red) and Hoechst for nuclear DNA (lower panel - blue). As can be seen, several cells with intensive GPA expression are decreased in size and have lost their nucleus (see details in figure legend). This result is correlated with typical terminal-stage of erythroid differentiation. The refined features of cell shapes as revealed by scanning electron microscopy (SEM) are shown in Figure 5C. The upper panel presents a typical ruffled immature K562 air-cell with a diameter of about 20 µm. For comparison, the lower panel presents a CO-cell, with typical differentiated and enucleated erythroid features such as concave, smooth shape and decreased size (7 µm in diameter). To further corroborate these observations, a FACS analysis of GPA expression dependence on cell size was performed on air- and CO-cells. The enclosed squares of Figure 5D demonstrates large fraction of small sized cells with increased GPA expression in CO-, but not in air-cells.

**Figure 6:**
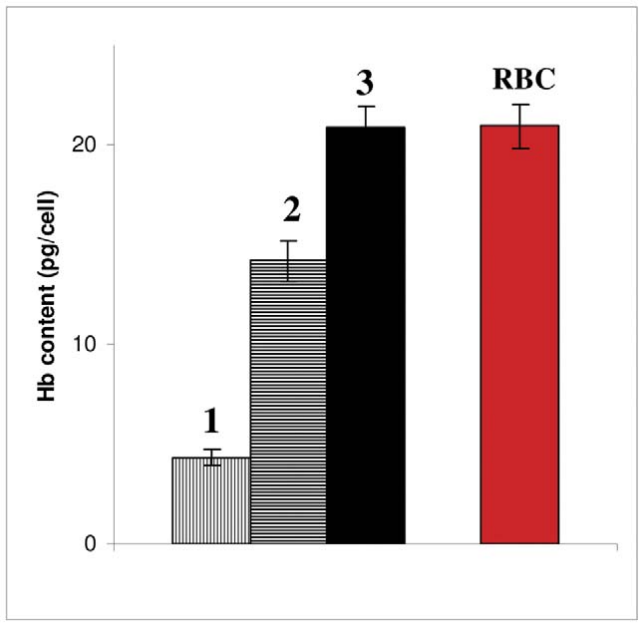
COA in enriched medium induces erythrocytes Hb synthesis. Cells were incubated for 3 days in regular or enriched medium (see Material and Methods for details). Hb content of cytosols was measured using “Hb quantification assay”. Compositions of media included: (**1**) medium only; (**2**) medium supplemented with “heme required nutrients”; (**3**) highly enriched medium; **RBC** - Hb content of mature erythrocytes.

To investigate whether the ability of CO to induce differentiation has been fully utilized, nutrient-enriched medium (required for Hb synthesis) was used. The results in Figure 6 demonstrate that under these conditions, Hb synthesis was significantly increased as compared to cell grown in a regular medium (Fig. 4C). As seen in bar 2 of Figure 6, addition of components required for heme synthesis resulted in elevated average Hb content. Additional enrichment with amino acids, required for globin synthesis, lead to a further increase in Hb content (bar 3 of Fig. 6). Interestingly, this combined enrichment enabled the cells to synthesize the amount of Hb which is normally present in mature red blood cell.

**Figure 7:**
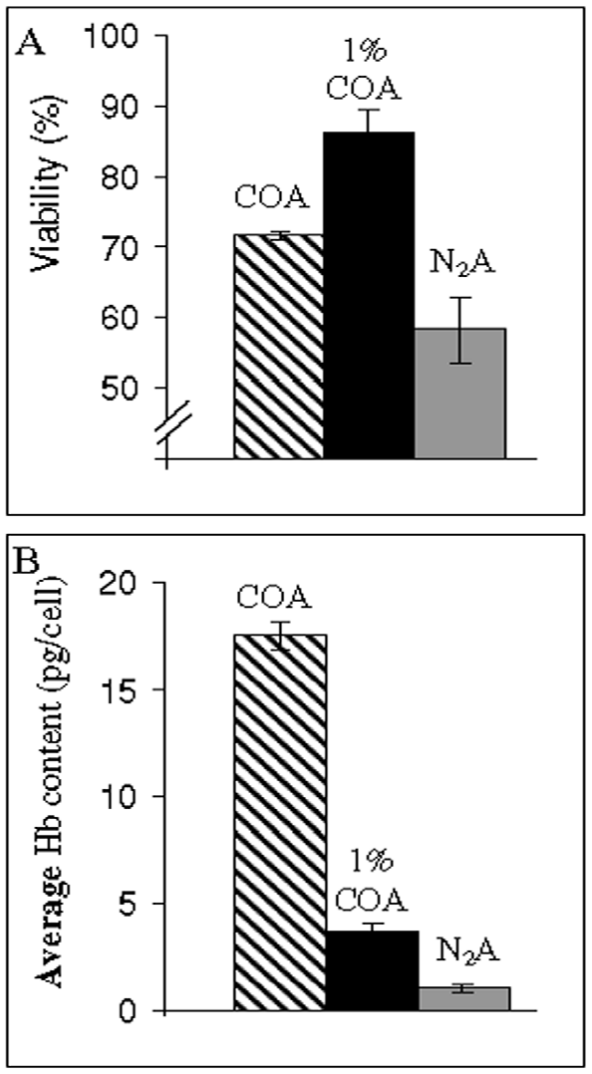
1%COA suffices for cell viability preservation and elevated average Hb content. Cells were incubated anaerobically with COA, N_2_A or 1% COA for 4 days in fully enriched media (details in Fig. 6, bar 3). Hatched bar - COA, black - 1% COA and grey - N_2_A. (**A**) Cell viability as assessed by TB assay. Note discontinuous Y-axis. (**B**) Average Hb content in cell population as measured by “Hb quantification assay” (see Material and Methods for details).

**Figure 8:**
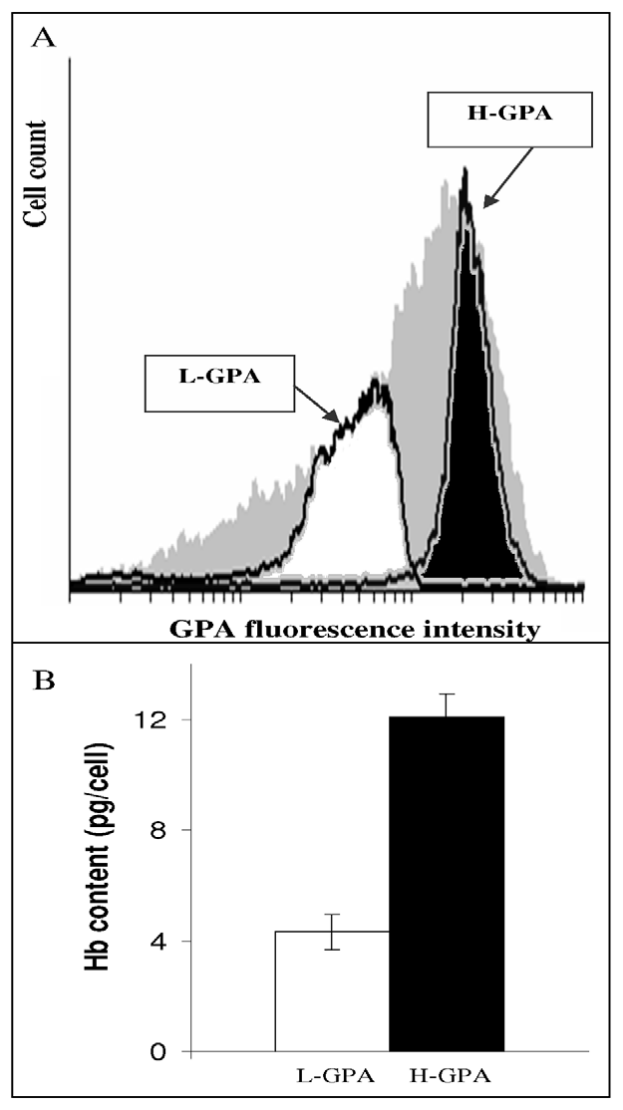
1% COA-cells contain a subpopulation with elevated Hb content. Cells were grown for 4 days in highly enriched medium under 1% COA and sorted by FACS for higher (H-GPA) and lower (L-GPA) GPA expression. (**A**) Grey area: GPA of total cell population. Black area: cells with H-GPA population. White area: cells with L-GPA population. (**B**) Hb content of sorted cells was measured by “Hb quantification assay” (see Material and Methods for details). White bar: L-GPA average Hb level. Black bar: H-GPA average Hb level.

Up to this stage, our results demonstrated that COA facilitates the induction of erythroid differentiation. However, these data were obtained under the non-physiological conditions of CO saturated atmosphere. Although not yet quantified, much lower CO concentrations may exist *in vivo*. Thus, the results of above experiments demonstrate the potential effects of CO at high concentration in the liquid phase, but may not be implemented to *in vivo* conditions in the BM. Therefore, further experiments were conducted to determine whether CO retains its specific differentiation effects even at a drastically reduced concentration. To test this assumption, additional studies were carried out comparing traits of cells kept under N_2_A or 1% COA (i.e. 99% N_2_ and 1% CO). We found, under all atmospheric and enriched medium conditions, that viability was preserved during the first 3 days of incubation (data not shown). At extended time periods (beyond 4 days), viability of N_2_-cells and CO-cells significantly decreased, while under 1% COA viability of most cells was preserved (Fig. 7A). Measurement of Hb content indicated that although 1% CO-cells were induced to synthesize Hb as compared to N_2_-cells, the average Hb concentration was much lower than that of COA cells (Fig. 7B). These findings could reflect either reduction of Hb concentration in all cells or an outcome of full differentiation of only a small cell fraction. Indeed, as shown in Figure 8A, the FACS analysis of 1% CO-cells (grey area) depicts a broad spectrum of GPA expression in this population. In order to distinguish between the two possibilities, cells were further separated into two sub-populations of higher (H-) and lower (L-) GPA expression, and the average Hb content of each fraction was quantified (Fig. 8B). As seen, Hb content of H-GPA expression cells was significantly higher than those of L-GPA. For technical reasons we could not sort cells with the highest GPA expression, which would probably have resulted in Hb content similar to that of mature erythrocytes. Taken together, the above findings confirmed that 1%COA induces heterogeneous erythroid maturation, resulting in cell populating the entire spectrum of differentiation stages.

## Discussion

The results of the current study reveal that CO is adequate for driving erythroid progenitor cells towards differentiation. This information assists a premise that CO is the necessary “nursing” material supplied by the central macrophage to developing erythroid progenitors in the EI.

Preservation of cell viability under COA anoxia is in agreement with the literature information even beyond cellular level. For example, *C. Elegans* embryos have been shown to conserve their animation following maintenance at COA [29]. Importantly, it appears that CO-induced erythroid differentiation (Fig. 4) is accompanied by cell cycle S-phase arrest (Fig. 3). The link between erythropoiesis and cell cycle arrest has been shown by a number of earlier studies indicating that S-phase progression is synchronized with several rapid committal differentiation stages [30]. Specifically, the erythroid transition from pro-erythroblast to basophilic normoblast is accompanied by S-arrest [31]. Under COA, enucleation of differentiated cells occurred (Fig. 5) and physiological erythrocyte Hb concentrations were obtained (Fig. 6) in the absence of macrophages. These findings are in accordance with previous studies where erythroid differentiation could be induced in absence of macrophages, but with reduced efficiency [3], [32].

To search for CO specific effects, the highest CO concentration at atmospheric pressure, which resulted in a water phase concentration of ∼1 mM , was used in the first part of the current study. Although at this stage, no precise information exists regarding CO concentrations in the BM, it is expected to be much lower. Thus, the second part of the study explored whether 1% CO in the atmosphere, with a resulting CO concentration of 10 µM in the water phase, was still capable of maintaining cell viability and inducing erythroid differentiation. Surprisingly, 1% CO in N_2_ atmosphere was sufficient to prevent cell death observed under N_2_A (Fig. 7A); moreover, 1% CO induced erythroid maturation. Under these anaerobic, low CO conditions, average Hb content was increased in comparison to N_2_A, but it was much lower than that of COA cells (Fig. 7B). Data analysis (Fig. 8) indicates that the low average Hb content was a result of diverse cellular sub-populations each at a different maturation stage. Thus, at low CO anaerobic atmospheric conditions, only a minor fraction of total cell population attains full differentiation, while other sub-populations reside at various lower maturation stages. Interestingly, this *in vitro* model distribution of cellular maturation closely parallels the wide spectrum of erythroblast maturation stages surrounding the central macrophage *in vivo* [1].

As noted above, erythroblasts lack HO-1 activity have decreased HO-2 expression during the terminal differentiation stage. Thus, it seems that the HO activity in the EI reside only in the central macrophage. Furthermore, these cells have long been recognized to retain high HO activity [33], which in context of the current study results, may affect erythropoiesis via CO production.

A key question arising from this study is whether the results contradict the well-known role of erythropoietin (EPO) as a key regulator of red cell production [34]. Probably not, since a link between EPO and HO-1 expression and function has become clear in recent years [35]–[36]. While EPO exerts its macro-effect via systemic pathways, HO-1/CO-induced differentiation seems to be limited to the EI microenvironment.

What are the possible heme sources to induce HO-1 activity in the EI central macrophage? As stated earlier, one well known source is Hb residues, phagocytized along with the engulfed nuclei. Another possible source could be direct transfer of excess toxic heme [37] through the tightly attached membranes of the two cell types. Recently, studies aimed at elucidating the mechanisms by which heme is cleared from erythroblasts membrane revealed that the trans-membrane protein, feline leukemia virus subgroup C receptor (FLVCR), acts as a heme exporter [38]. The expression of FLVCR is increased in human BM erythroid precursors during the time course of erythropoiesis, where its expression peaks at the intermediate stage of maturation and wanes at terminal maturation stages [10]. This observation suggests that FLVCR is uniquely important for CFU-E-proerythroblast survival and/or differentiation [39]. For efficient clearance of the FLVCR attached heme, further steps of heme transfer from FLVCR and its disintegration are required. Indeed one such option of heme transfer from FLVCR to hemopexin was shown, where the heme bound hemopexin is further cleared in the liver [40]. An alternative clearance option, specific to EI structures, could be the capture of FLVCR bound heme by the HO-1, located in the macrophage plasma membrane [41], a step well suited to the stage of terminal erythroid differentiation, where the two cell type membranes are tightly attached. This pathway could facilitate increased CO production, which in turn diffuses back into the attached erythroblasts to trigger additional erythroid maturation.

**Figure 9:**
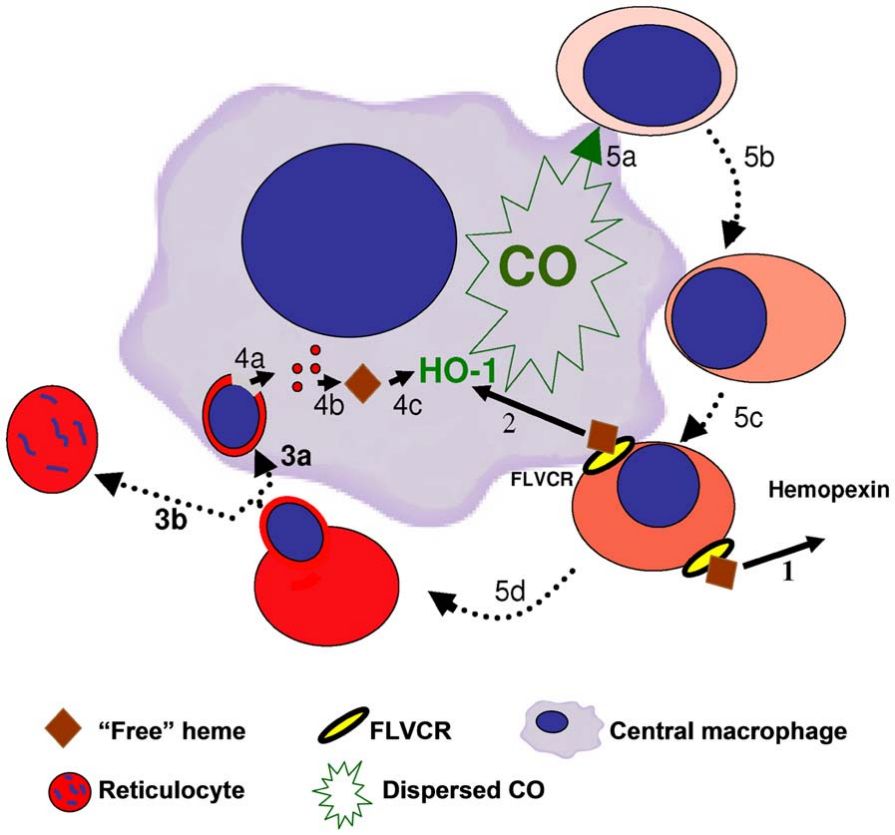
Schematic presentation of CO production and function in the erythroblastic island. **Shaded Red** - Hb at varying concentrations; **Blue** – nucleus; **Green** – CO. Stages of differentiation in EI erythroblasts are shown in a clockwise fashion. 1: Clearance of FLVCR-associated “free” heme by hemopexin. 2: HO-1 induction by FLVCR associated “free” heme. 3: Enucleation of terminally differentiated erythroblast in EI. 3a: Engulfment of nucleus containing Hb remnants. 3b: Reticulocyte movement toward blood circulation. 4: HO-1 induction by Hb heme remnants. 4a. Dispersion of Hb in central macrophage. 4b. Formation of “free” heme from Hb phagocytosed with nucleus. 4c: Induction of HO-1 by “free” heme. (The membrane attached HO-1 is shown in the macrophage center for illustration purpose only.) 5: CO production by HO-1, leading to terminal differentiation. 5a. CO dispersion reaching erythroblast. 5b. Nuclear condensation and peripheral shift. 5c. Accelerated Hb synthesis, followed by membrane-associated “free” heme. 5d. Terminal erythroid differentiation upon attaining maximal Hb content.

In conclusion, the results of the current study indicate that HO-1-produced CO could be the “nursing” material provided by the central macrophage to stimulate reticulocytes production in the EI. Figure 9 represents a schematic model integrating the results of the current study, and illustrating the various pathways connecting erythroid differentiation and CO production. Further investigations are required to clarify the details of this process on a molecular level.
